# Study of Direct-Contact HfO_2_/Si Interfaces

**DOI:** 10.3390/ma5030512

**Published:** 2012-03-19

**Authors:** Noriyuki Miyata

**Affiliations:** National Institute of Advanced Industrial Science and Technology (AIST), Tsukuba, Ibaraki 305-8562, Japan; E-Mail: nori.miyata@aist.go.jp; Tel.: +81-29-861-2511; Fax: +81-29-861-2576

**Keywords:** MOSFET, high-*k*, HfO_2_, interface dipole, channel mobility

## Abstract

Controlling monolayer Si oxide at the HfO_2_/Si interface is a challenging issue in scaling the equivalent oxide thickness of HfO_2_/Si gate stack structures. A concept that the author proposes to control the Si oxide interface by using ultra-high vacuum electron-beam HfO_2_ deposition is described in this review paper, which enables the so-called direct-contact HfO_2_/Si structures to be prepared. The electrical characteristics of the HfO_2_/Si metal-oxide-semiconductor capacitors are reviewed, which suggest a sufficiently low interface state density for the operation of metal-oxide-semiconductor field-effect-transistors (MOSFETs) but reveal the formation of an unexpected strong interface dipole. Kelvin probe measurements of the HfO_2_/Si structures provide obvious evidence for the formation of dipoles at the HfO_2_/Si interfaces. The author proposes that one-monolayer Si-O bonds at the HfO_2_/Si interface naturally lead to a large potential difference, mainly due to the large dielectric constant of the HfO_2_. Dipole scattering is demonstrated to not be a major concern in the channel mobility of MOSFETs.

## 1. Introduction

High-*k* HfO_2_ gate dielectrics have been employed in state-of-the-art complementary metal-oxide-semiconductor field-effect-transistors (CMOSFETs), which have contributed to improved performance of devices and reduced power consumption [1,2]. Before the beginning of the 1990s, thermally grown SiO_2_ was the only gate dielectric employed in the mass production of CMOSFETs [3]. Although small amounts of nitrogen had then been incorporated into SiO_2_ to prevent dopant diffusion from the poly-Si gate, the SiO_2_/Si interface has continuously been utilized because of its excellent interface characteristics, *i.e.*, low interface state and low fixed charge densities. When the physical thickness of SiON layers was scaled down below 2 nm in the mid-1990s, power consumption due to direct-tunneling gate-leakage current was highlighted, and research on high-*k* technology started throughout the world [4].

Various metal oxides, e.g., ZrO_2_, Al_2_O_3_, Y_2_O_3_, and La_2_O_3_, were studied as candidates for high-*k* materials when research began [4,5,6,7,8], and HfO_2_ was then finally employed in mass production [1,2]. The author of this paper has focused on the fundamental characteristics of the HfO_2_/Si system until the early 2000s. In particular, the growth kinetics of the low-dielectric-constant layer at the HfO_2_/Si interface has intensively been investigated, as its low dielectric constant (SiO_2_ ~3.9) obviously results in disadvantages in scaling the equivalent oxide thickness (EOT) down to less than 1 nm [4]. For example, it has been predicted that the interface Si oxide layer should be suppressed to 1–2 monolayers (~0.3–0.5 nm) to achieve an EOT of ~0.7 nm. From the viewpoint of EOT scaling, the direct-contact HfO_2_/Si interface is an optimum structure. However, Hf-Si bonds are not suitable as they may have electric states in the Si band gap [9,10]. Thus, the Si surface should be terminated at least by one monolayer of oxygen (and/or hydrogen), *i.e.*, monolayer Hf-O-Si bonding is an optimum interface structure. However, since it is difficult to control interface Si oxide under standard process conditions, e.g., atomic layer deposition and chemical vapor deposition, the HfO_2_/SiO_2_/Si stack structure has been employed in practical device development [11,12].

As continuous EOT scaling has been required in the development of advanced MOSFETs [13], I felt that it was valuable to challenge the preparation of a direct-contact HfO_2_/Si structure. However, Si oxidation under the HfO_2_ layer has not been very controllable because of the so-called catalytic effect by HfO_2_, e.g., the small amount of residual oxygen and water in the deposition and post-deposition annealing (PDA) ambient immediately produces several monolayers of Si oxide layers [14,15,16], meaning that low O_2_ pressure conditions should be applied. However, a certain amount of oxygen pressure is required to reduce defects in the HfO_2_ layer (e.g., oxygen vacancies). Therefore, we should find appropriate O_2_ pressure conditions that are compatible with slow interface Si oxidation and low defect density in HfO_2_.

Ultra-high vacuum electron-beam (UHV-EB) HfO_2_ deposition and *in-situ* post-deposition annealing (PDA), which enable the preparation of direct-contact HfO_2_/Si structures, are described in this article. The HfO_2_/Si structures demonstrate low interface state densities but unexpected strong interface dipoles. The effect of interface dipoles on the channel mobility of MOSFETs is also discussed.

## 2. Electron-Beam HfO_2_ Deposition

**Figure 1:**
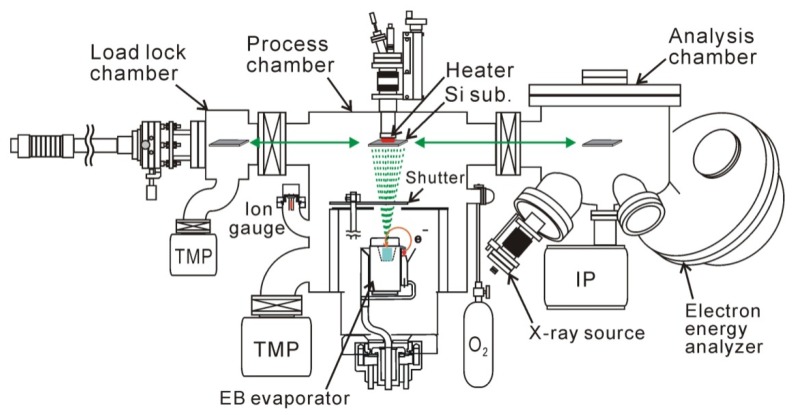
Experimental setup used for HfO_2_ deposition, post-deposition annealing (PDA), and surface analysis. XPS system was connected to UHV-EB evaporation chamber for *in-vacuum* analysis of surface chemistry.

We should carefully choose process conditions by taking into account fundamental reactions in the HfO_2_/Si system. Interface layer growth and silicidation are major reactions taking place during HfO_2_ deposition and PDA. The former is particularly important in EOT scaling. There have been numerous reports on the growth of interface layers; the major chemical components have been ascribed to be SiO_2_, although small numbers of Hf atoms have sometimes been included (*i.e.*, Hf silicate) [14,15,16,17,18]. The fact that Si oxide was formed implies that the Si surface was oxidized during HfO_2_ deposition and PDA. Thus, it is important to suppress the oxygen that is supplied to the Si surface from the process ambient, even though a certain amount of oxygen is required to produce Hf oxide. Therefore, we needed to carefully examine a wide range of O_2_ pressures to find optimum process conditions. UHV-EB evaporation is suitable for such experiments, because we can easily control the oxidant (O_2_) pressure in the high-vacuum range. A metallic Hf source was evaporated under an O_2_ pressure of 10^−4^–10^−8^ torr (Figure 1) in our experiment. This deposition setup had a load lock chamber and a surface analysis system that involved x-ray photoelectron spectroscopy.

Three Si-oxidation stages should be taken into account at least in the current processes: (1) Si oxidation before HfO_2_ deposition; (2) Si oxidation during HfO_2_ deposition; and (3) Si oxidation during PDA. Controlling the third stage is particularly serious, as the oxidation rate is much faster due to the HfO_2_ catalytic effect and the raised sample temperature (>400 °C) [16]. This issue will be discussed in the next section. This section focuses on the first and second stages. A hydrogen-terminated Si (100) surface formed by diluted hydrofluoric (HF) acid solution was used as the initial surface, because it is stable under atmospheric conditions [19]. However, this Si surface is not very stable in a vacuum chamber, e.g., a filament of the ion gauge dissociates residual molecules to break H-Si bonds on the Si surface. The sample in this work was slowly evacuated in the load-lock chamber, and it was then transferred to the deposition chamber without turning on the ion gauge in the load-lock chamber. The *in-vacuum* x-ray photoelectron measurements revealed that the surface coverage of oxygen on the Si substrate was less than 5%.

A certain amount of oxygen should intentionally be introduced into the chamber to produce metal oxide in the process of HfO_2_ deposition. We can easily predict that oxygen molecules will be dissociated by the filament of the ion gauge and of the EB evaporator as well as by the heated Hf source. The dissociated species immediately oxidizes the surface Si before and during HfO_2_ deposition. Thus, the ion gauge was carefully placed to prevent direct incidence of hot-filament-dissociated species onto the Si surface. The EB evaporator was separated from the sample with a water-cooled housing and shutter. The HfO_2_ was deposited at about 0.2 nm/min under an O_2_ pressure of 2 × 10^−6^ torr. The sample heater was turned off to suppress Si oxidation. Although radiation from the EB evaporator slightly raised the sample temperature, this was confirmed to be less than 100 °C by thermocouple measurement of the sample surface. The Si 2p photoelectron spectra revealed that the Si oxide was less than 0.15 nm thick after HfO_2_ deposition, suggesting the interface Si-O bonds were less than one monolayer [20]. The Hf 4f photoelectron spectra exhibited stoichiometric HfO_2_, even though the deposition was carried out under such low O_2_ pressure and low temperature conditions.

Although the XPS revealed stoichiometric HfO_2_, small numbers of defects were likely formed in the as-deposited HfO_2_. The MOS capacitors of the HfO_2_/Si structure actually showed unusual *C-V* curves and hysteresis. Therefore, PDA was required to eliminate the defects. Note that if the sample was exposed to air before PDA, fixed positive charges were created in HfO_2_ due to the reaction between defects with moisture [21,22]. This type of electric charge can be eliminated by using PDA, but high temperature annealing is required (>600 °C). Thus, *in-situ* PDA in the EB deposition chamber was performed in this experiment.

## 3. Kinetics of Interface Si Oxidation in PDA

As was described in the last section, interface Si-O bonds could be suppressed to less than one monolayer by employing UHV-EB deposition HfO_2_ method. Searching for optimum PDA conditions was more complicated, because of immediate Si oxidation due to the HfO_2_ catalytic effect and local silicidation. The Si oxide layer increased in thickness with increasing temperature and O_2_ pressure, while the annealing time was fixed (~several minutes) [16]. Under low O_2_ pressure conditions (<10^−6^ torr), on the other hand, local silicidation took place at temperatures higher than 800 °C [16]. Atomistic mass transfer on the HfO_2_ surface led to local thinning of the HfO_2_ layer, and this resulted in the formation of voids [23,24]. Hf silicide was formed in these voids due to the reaction of HfO_2_ with mobile Si atoms on the void surfaces (HfO_2_ + 4Si → HfSi_2_ + 2SiO↑) [25]. Since Hf silicide is metallic, MOS devices that underwent this silicidation did not work. The maximum PDA temperature that can suppress silicidation depends on the duration of PDA, as void formation is a time-development phenomenon. For typical durations of several minutes, we should chose a PDA temperature that is below 800 °C to suppress silicidation.

**Figure 2:**
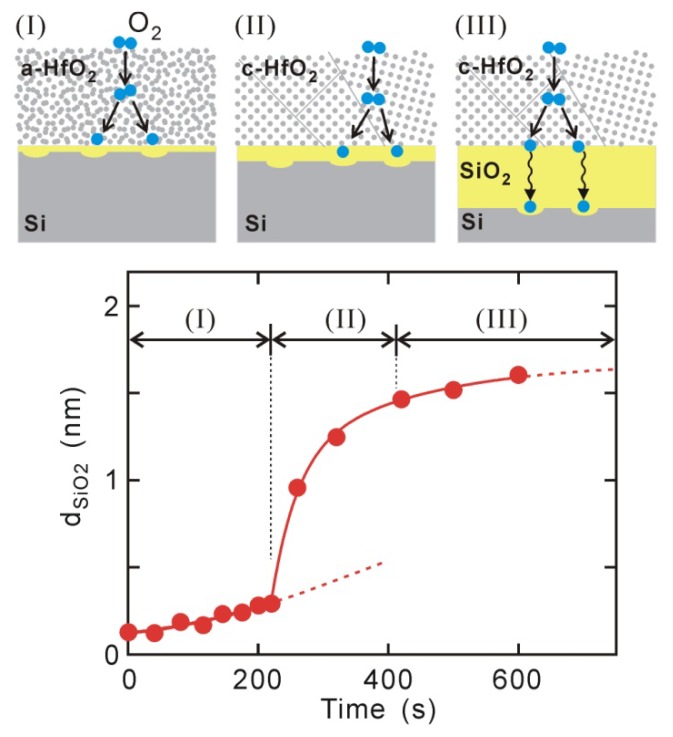
Si oxidation at 1.5-nm HfO_2_/Si interface during PDA in O_2_ pressure of 2 × 10^−6^ torr at 400–500 °C. Oxidation occurred in three steps of (**I**) slow oxidation in range of monolayer Si oxide; (**II**) rapid oxidation up to thick SiO_2_ layer exceeding 1 nm; and (**III**) slow oxidation demonstrating saturated behavior. Oxidation mechanisms expected for stages (I)–(III) have been depicted.

The thicknesses of Si oxide layers observed by XPS for the 1.5-nm-thick HfO_2_/Si structures are plotted in Figure 2, where PDA was performed at an O_2_ pressure of 2 × 10^−6^ torr at 400–500 °C. Si oxidation occurs in three stages: (I) slow oxidation in the monolayer range, (II) rapid oxidation up to thick SiO_2_ layers that exceed 1 nm, and (III) again slow oxidation showing saturated behavior. This indicates that PDA should be finished within stage (I) to maintain a direct-contact HfO_2_/Si interface. The activation energy for the first stage was estimated to be 0.3 eV, *i.e.*, the dependence on temperature was very weak [20]. This characteristic acts as an advantage from the viewpoint of controlling interface Si oxidation. The PDA in this work was thus conducted under these conditions, *i.e.*, an O_2_ pressure of 2 × 10^−6^ torr at 400–600 °C for several minutes.

Next, I will briefly explain why the mode of Si oxidation changes so dramatically during PDA. Although the oxidation rate in the first stage is smaller than those in the other stages, it is slightly higher than that on a clean Si(100)-2 × 1 surface. This means that atomic oxygen produced by defects in the as-deposited HfO_2_ layer contributes to Si oxidation even in the first stage. The crystallization of HfO_2_ in the second stage is responsible for the change in the mode of oxidation. The TEM observations actually revealed that a monoclinic HfO_2_ phase appeared in the second stage, although an amorphous HfO_2_ phase was maintained in the first stage [20]. This suggests that O_2_-dissociation sites, *i.e.*, defects (e.g., oxygen vacancies), are largely created by crystallization. As oxygen diffusion in the third stage through the grown interface SiO_2_ layer limited the whole oxidation process, the oxidation rate was saturated. The activation energy in this stage has been reported to be 0.72 eV, which is smaller than the 1.24 eV reported for O_2_ diffusion through the SiO_2_ layer [26,27]. This means that atomic oxygen is a major oxidant species in Si oxidation in the HfO_2_/Si system.

According to the above results, suppressing HfO_2_ crystallization is crucial to control interface Si oxide in the monolayer range. It has been reported that Al-, Si-, or N-incorporated HfO_2_ has an advantage in suppressing the growth of the interface layer. This is likely related to crystallization, *i.e.*, the incorporation of such atoms is expected to increase the temperature of crystallization. Furthermore, the insertion of an oxygen-diffusion-barrier layer into the HfO_2_/Si system e.g., Al oxide or Si nitride, effectively suppresses Si oxidation [26,28]. However, as their dielectric constants are lower than that of HfO_2_, these multi-stack structures are not suitable for achieving an EOT of ~0.5 nm. Incorporating additional atoms or an oxygen-diffusion-barrier layer is probably required while the sample undergoes high-temperature PDA (>600 °C). A simple HfO_2_/Si structure can be applied for fabricating a direct-contact HfO_2_/Si structure in low-temperature PDA (≤600 °C).

## 4. MOS Electrical Characteristics

As previously described, UHV-EB HfO_2_ deposition and *in-situ* PDA enabled a direct-contact HfO_2_/Si structure with stoichiometric HfO_2_ to be prepared and interface Si-O bonds of about one monolayer to be attained. The electrical characteristics of MOS capacitors are reviewed in this section. It has been reported that the EOT values for direct-contact 2.2-nm-thick HfO_2_/n-Si(100) MOS capacitors have been estimated to be 0.45–0.48 nm from their high-frequency *C-V* curves [20]. The dependence of EOTs on HfO_2_ thickness has indicated that the dielectric constant of the HfO_2_ layer is about 20. This value is approximately consistent with that of bulk HfO_2_, supporting the formation of a stoichiometric HfO_2_ layer [4]. The dependence on thickness has also supported the argument that the interface layer is about zero for MOS capacitors fabricated on n-type Si substrates. However, the accumulation capacitances of MOS capacitors on p-type Si substrates were slightly smaller than those on n-type Si substrates, where additional layers with an EOT of ~0.26 nm virtually existed at HfO_2_/Si interfaces [29]. However, XPS observations did not reveal any differences in the interface Si oxide. This unexpected reduction in accumulation capacitance at the p-Si substrate could be explained based on the effect of the interface dipole, *i.e.*, the hole distribution for the p-Si surface is slightly shifted far from the HfO_2_/Si interface due to the electrostatic potential of the interface dipole. We should note that this effect acts as a disadvantage in the operation of p-channel MOSFETs.

Interface state density (*D_it_*) can roughly be inferred from the frequency dispersion in *C-V* curves. The *C-V* frequency dispersion of direct-contact HfO_2_/Si MOS capacitors has suggested that *D_it_* near the mid-gap energy is on the order of 10^11^ cm^−2^eV^−1^ or less. A method of ac-conductance, which is more accurate, actually indicates a *D_it_* of ~10^11^ cm^−2^eV^−1^ at around the mid-gap energy [30]. This is approximately equal to those of HfO_2_/SiO_2_/Si stack structures prepared with the same methods of UHV-EB and *in-situ* PDA. The MOSFET characteristics also support the small *D_it_*, as will be explained in what follows. Thus, we can conclude that Si dangling bonds at the direct-contact HfO_2_/Si interface are effectively suppressed even though interface Si-O bonds are in the range of about one monolayer.

**Figure 3:**
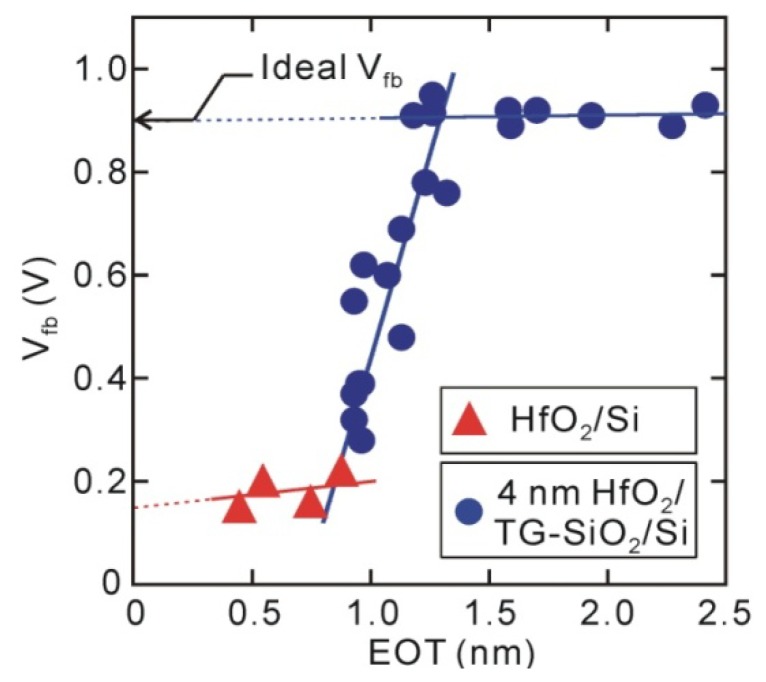
*V_fb_* for direct-contact HfO_2_/Si and 4 nm HfO_2_/TG-SiO_2_/Si MOS capacitors. Negative *V_fb_* shift for direct-contact HfO_2_/Si MOS capacitor is mitigated by insertion of thin Si oxide layer (~0.5 nm).

Next, let us discuss the distribution of fixed electric charges in the direct-contact HfO_2_/Si structures from the behavior of flat-band voltages (*V_fb_*). The direct-contact samples demonstrated the obvious negative *V_fb_* shifts shown in Figure 3, and their values did not depend on the HfO_2_ thickness. Unipolar-electric charges that exist at the oxide/Si interface usually show a *V_fb_* shift that is dependent on oxide thickness, but dipole-like charges show *V_fb_* shift that is independent of thickness [31]. Thus, it has been concluded that direct-contact HfO_2_/Si MOS capacitors have a strong interface dipole (>0.5 V, positively charged Si and negatively charged HfO_2_ sides) [32]. However, the HfO_2_/SiO_2_/Si stack structures with thick interface SiO_2_ layers have almost ideal *V_fb_*. The *V_fb_* behavior observed for the HfO_2_/thickness-graded (TG) SiO_2_/Si structure in Figure 3 reveals that negative *V_fb_* shift in the direct-contact samples is almost completely mitigated by inserting an SiO_2_ layer that is about 0.5 nm thick, meaning that the interface dipole was eliminated by inserting an additional Si-O layer that was 3–4 monolayers. Thus, an interface dipole observed for the direct-contact HfO_2_/Si structure presumably exists at the HfO_2_/Si interface. Similar interface dipoles that are larger than 0.5 V have been observed from direct-contact HfO_2_/Ge structures that have interface Ge-O bonds of about one monolayer [33].

**Figure 4:**
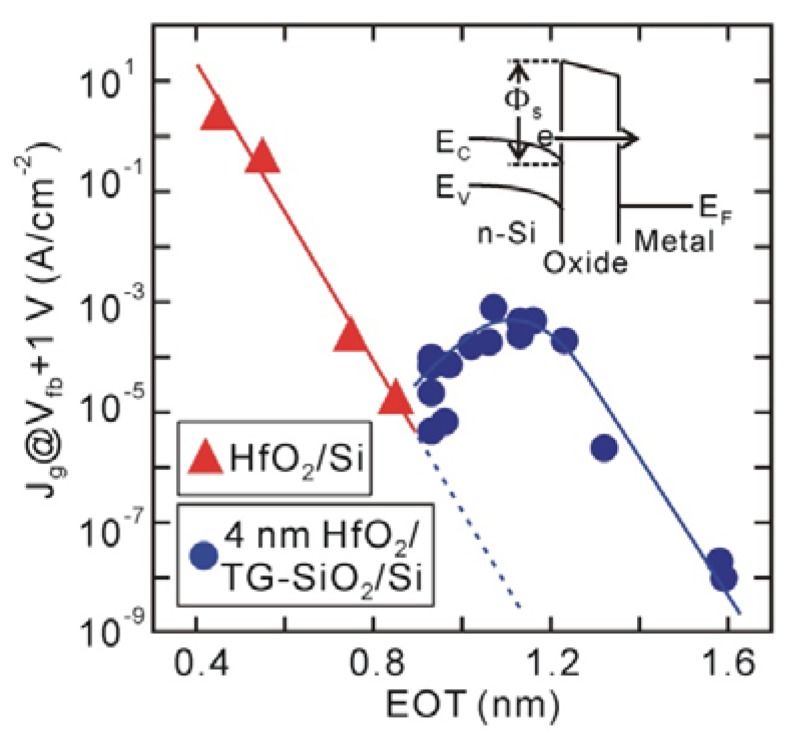
*J_g_* at *V_fb_* + 1 V for direct-contact HfO_2_/Si and 4 nm HfO_2_/TG-SiO_2_/Si MOS capacitors fabricated on n-type Si substrates. *J_g_* for HfO_2_/TG-SiO_2_/Si MOS capacitors increases with increasing Si oxide layer because of reduced conduction band offset (*Φ_s_*) at HfO_2_/Si interface.

The leakage current (*J_g_*) characteristics also support the existence of an interface dipole (Figure 4). Generally, *J_g_* exponentially decreases with increasing dielectric layer thickness, while trap-assisted current is sufficiently suppressed, as those of the direct-contact HfO_2_/Si structures in Figure 4 show. This means that both conduction band offset (*Φ_s_*) at the HfO_2_/Si interface and HfO_2_ thickness govern electron tunneling. Since the conduction band offset of the SiO_2_/Si interface (*Φ_s_* ~3.15 eV) [34] is larger than that of the HfO_2_/Si interface (*Φ_s_* ~1.1–1.5 eV) [35,36,37], the leakage current is expected to decrease by inserting an Si oxide layer, as indicated by the dotted line in Figure 4. However, the *J_g_* of HfO_2_/TG-SiO_2_/Si MOS capacitors increases once and then decreases, probably meaning that the barrier height is reduced by the insertion of an interface Si oxide. In addition, the *Φ_s_* values at the direct-contact HfO_2_/Si interfaces were estimated to be about 2.0 eV, which were obtained by fitting the measured *J_g_* curves with a direct-tunneling model. This estimated barrier height is larger by at least 0.5 eV than those previously reported for HfO_2_/Si systems [35,36,37], suggesting that the conduction band offset at the direct-contact HfO_2_/Si interface is raised by the interface dipole [32].

## 5. Origin of Interface Dipole

This section discusses the physical origin as to why a strong interface dipole was formed in direct-contact HfO_2_/Si MOS capacitors. First, we should examine extrinsic effects, e.g., the fabrication process for metal gates induces electric charges in gate stack structure [38,39]. As-prepared HfO_2_/Si samples should be evaluated with some surface analysis without gate metal layers being deposited to investigate such effects. X-ray photoelectron spectroscopy (XPS) is a potential method, which has often been used to identify the band lineups of oxide/semiconductor systems [40,41]. Our group employed hard x-ray photoemission spectroscopy as well as conventional XPS (Al k*α* x-ray source) to compare metal/HfO_2_/Si and HfO_2_/Si structures, but we could not find clear evidence that supported the existence of an interface dipole, because x-ray-induced electrical charges and screening effects around the HfO_2_/Si interface hampered band lineups from being accurately estimated [42]. Thus, Kelvin probe measurements, which are not accompanied by the creation of electrical charges in the oxide, were used to explore the origin of dipole formation.

**Figure 5:**
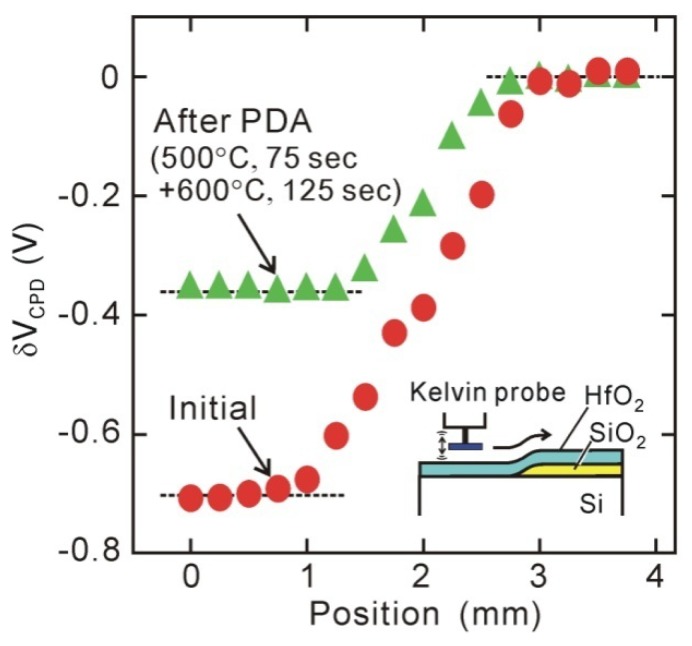
*In-situ* Kelvin probe measurements for normalized contact potential difference (*δV_CPD_*). Large *δV_CPD_* difference between direct-contact HfO_2_/Si and HfO_2_/SiO_2_/Si stack structures indicates existence of interface dipole.

The Kelvin probe measurement is a conventional surface analysis, which has widely been used to measure the work function of metal surfaces. A reference electrode was placed on the sample surface with a small air or vacuum gap (0.1–0.5 mm). The alternating current induced by vibrating the reference electrode was monitored to determine the contact potential difference (CPD) between the sample metal and reference electrode surfaces. The surface carrier response should be taken into account for the semiconductor surface, but an n-type semiconductor surface can be analyzed like a metal surface provided that the response of holes can be neglected. The band diagram for the oxide/Si surface is quite similar to that obtained from MOS measurements except for the vacuum gap [43,44]. Thus, we can infer the distribution of electric charges inside the oxide/Si structure from the behavior of *V_CPD_*. Figure 5 plots the normalized CPD voltage (*δV_CPD_*) observed from the sample surface that includes both direct-contact HfO_2_/Si and HfO_2_/SiO_2_/Si stack structures, which indicates that the surface potential of the direct-contact HfO_2_/Si area is smaller by about 0.7 V than that of the HfO_2_/SiO_2_/Si stack area [44,45]. This potential difference is approximately consistent with the negative *V_fb_* shifts of MOS capacitors. This means that the interface dipole is formed before the metal gate is fabricated.

**Figure 6:**
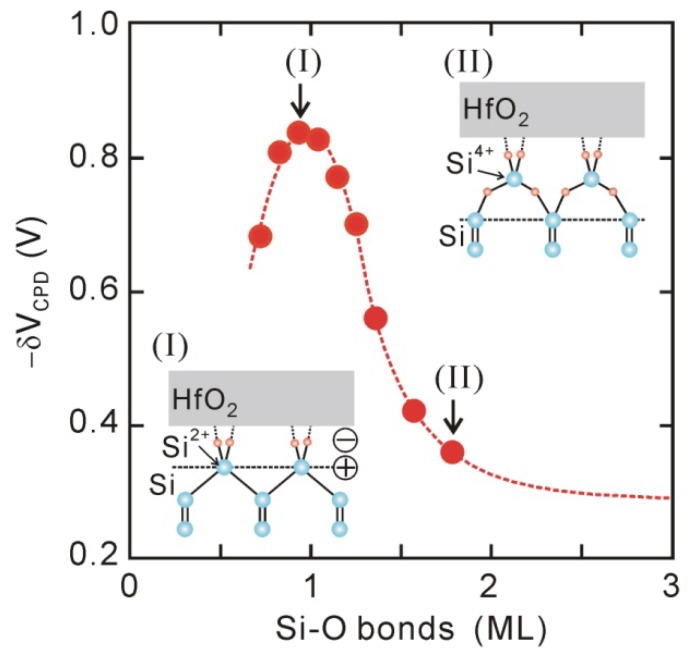
*δV_CPD_* for direct-contact HfO_2_/Si structures after various PDA processes, as a function of Si-O thickness estimated by *in-vacuum* XPS.

*In-situ* Kelvin probes and XPS have been used to assess the effect of interface chemical bonding on the interface dipole. Figure 6 plots the *δV_CPD_* values for direct-contact HfO_2_ (1.5 nm)/Si structures that underwent various PDA in the first oxidation stage in Figure 2, as a function of the thickness of the Si-O layer. The dipole strength becomes maximal at Si-O bonds of about one monolayer, and then decreases in the range of Si-O bonds of 1–2 monolayers. This behavior means that Si-O bonds of one monolayer are responsible for the interface dipole, and inserting oxygen atoms into the back bonds of these surface Si atoms results in the dipole being annihilated as shown in the insets in Figure 6. In addition, it has been reported that the interface dipole completely disappears while the interface Si-O bonds thermally decompose in UHV [45]. Similar annihilation of the dipole could be found for direct-contact HfO_2_/Ge structures [33]. Thus, we can conclude that the Si-O bonds of about one monolayer at the HfO_2_/Si interface mainly induce the interface dipole [44,45].

The formation of an interface dipole has been a long-standing controversy in the engineering of band lineups in metal/semiconductor, semiconductor/semiconductor, and oxide/semiconductor systems. Various mechanisms have been proposed, which can be roughly classified into two models. The first is charge transfer due to interface states, e.g., metal-induced-gap states (MIGS) and dielectric-constant-induced gap states (DCIGS) [46,47,48,49]. The second is based on the electric polarization of interface chemical bonds [50,51,52]. The *C-V* and ac-conductance measurements revealed that charge trapping at the direct-contact HfO_2_/Si interface is not serious as previously mentioned [30], so that the contribution of the first model is not as large in this case. Thus, the second model is preferable, as the Kelvin probe measurements demonstrated. However, it has widely been accepted that SiO_2_/Si structures, which are prepared by using appropriate thermal oxidation processes, do not have such strong dipoles, even though there are Si-O bonds at this interface. The author’s group thus compared electrostatic potential between atomistic models of SiO_2_/Si and direct-contact HfO_2_/Si interfaces [44]. As for the SiO_2_/Si interface, the potential on the SiO_2_ side is expected to be close to that on the Si side, as the potential drop by the interface Si-O bonds is compensated by the potential rise by the upper O-Si bonds. However, the potential rise by the O-Hf bonds in the direct-contact HfO_2_/Si interface was estimated to be smaller than that by the O-Si bonds in the SiO_2_/Si interface, as the dielectric constant for the O-Hf region was expected to be larger than that for the O-Si region. We, therefore, concluded that a dipole formation is a natural phenomenon for the atomically abrupt high-*k*/Si interface having one-monolayer Si-O bonds.

## 6. MOSFET Characteristics and Channel Mobility

**Figure 7:**
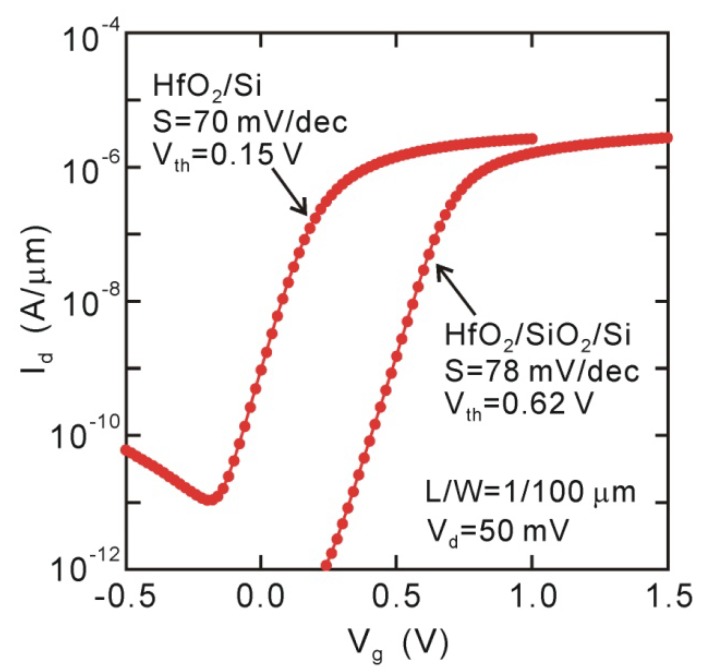
*I_d_-V_g_* curves of 2.5 nm HfO_2_/Si and HfO_2_/SiO_2_/Si MOSFETs. Large negative *V_th_* shift takes place for direct-contact HfO_2_/Si device.

As previously described, direct-contact HfO_2_/Si structures have an advantage in EOT scaling in ranges thinner than 1 nm. The interface state density could be suppressed to ~10^11^ cm^−2^eV^−1^ at around the midgap, probably because of surface termination with Si-O bonds of about one monolayer. Thus, we could expect MOSFETs to work well except for the effect of the interface dipole. The *I_d_*-*V_g_* curves for the direct-contact HfO_2_/Si MOSFET in Figure 7 actually indicate good behavioral characteristics. The *I_on_/I_off_* ratio is larger than 10^5^, even though a very thin HfO_2_ layer of 2.5 nm was used in the direct-contact HfO_2_/Si structure (EOT ~0.52 nm) [20]. The subthreshold slope (*SS*) was almost independent of the interface structures (*SS* ~70 mV/decay), which is approximately consistent with the *D_it_* value estimated with the ac-conductance measurement of MOS capacitors. Obvious negative *V_th_* shift can be identified in Figure 7, as predicted from the interface dipole observed for the MOS capacitors.

**Figure 8:**
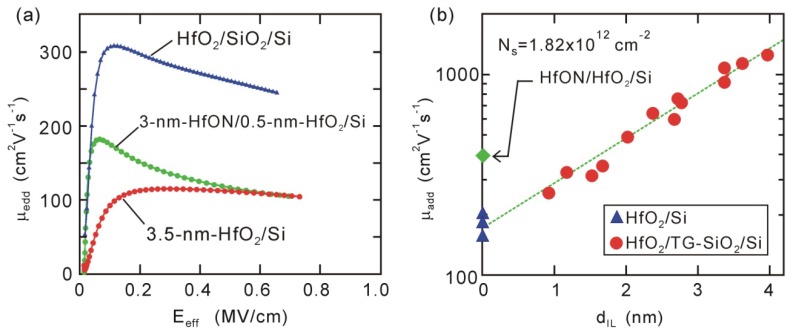
Electron mobility characteristics of HfO_2_/Si and HfON/HfO_2_/Si MOSFETs. (**a**) Effective mobility characteristics; and (**b**) additional electron mobility (*μ_add_*@*N_s_* = 1.82 × 10^12^ cm^−2^) for 3.5-nm HfO_2_/TG-SiO_2_/Si and 3-nm HfON/0.5-nm HfO_2_/Si MOSFETs.

The impact of interface dipoles on channel mobility has been pointed out in several papers, e.g., high-*k*/Si and high-*k/*III-V-channel MOSFETs [53,54,55]. Actually, electron mobility of direct-contact HfO_2_/Si MOSFET is smaller than that of HfO_2_/SiO_2_/Si stack MOSFET, as shown in Figure 8(a). Thus, we have examined what effect such an interface dipole has on the channel mobility of direct-contact HfO_2_/Si MOSFETs. The channel mobility of high-*k*-gated MOSFETs generally gradually decreases with thinning interface Si oxide layers (IL) due to the so-called remote scattering, *i.e.*, high-*k* phonon scattering and Coulomb scattering due to oxide charges in the high-*k* layer [56,57,58,59]. We should take such effects into account to identify the true impact of dipoles. Actually, additional electron mobility (*µ_add_*) decreases exponentially throughout the whole *t_IL_* range including direct-contact HfO_2_/Si interfaces, as seen in Figure 8(b). This tendency suggests that remote scattering is the dominant mechanism [56,57,58,59]. The variable temperature measurements suggested that remote scattering could mainly be ascribed to Coulomb effects due to oxide charges in the HfO_2_ layer [60]. The incorporation of nitrogen into HfO_2_ effectively reduced the oxide charges, as the HfON/HfO_2_/Si stack device had larger channel mobility than standard HfO_2_/Si device [Figure 8(a,b)].

If the dipole had some impact, a dramatic change should have been observed in the dependence of *µ_add_* on *t_IL_*, as the negative *V_fb_* and *V_th_* shifts were mitigated by inserting a ~0.5 nm thick Si oxide layer. It is obvious that the data observed in Figure 8(b) do not demonstrate such a dramatic change. In addition, the *µ_add_* of direct-contact HfO_2_/Si devices depended on the HfO_2_ thickness [60], although the dipole strength was nearly independent of the HfO_2_ thickness [32]. Thus, we can conclude that dipole scattering is not the dominant mechanism responsible for degradation in direct-contact HfO_2_/Si devices. We can expect that well-ordered dipoles, which do not produce large electrostatic potential fluctuations, do not act as strong scattering centers, e.g., interface chemical bonds at lattice-matched heterojunctions do not induce serious degradation in mobility [61]. The present results suggest that uniformly arrayed dipoles are formed at direct-contact HfO_2_/Si interfaces. This speculation is consistent with the above dipole model based on the electrostatic potential of interface Si-O and O-Hf bonds [44,45].

## 7. Conclusions

UHV-EB HfO_2_ deposition and *in-situ* PDA enabled us to prepare the so-called direct-contact HfO_2_/Si structure. The electrical characteristics of direct-contact HfO_2_/Si MOS capacitors demonstrated low interface state density but a strong interface dipole. The *in-situ* Kelvin probe measurements of HfO_2_/Si structures provided clear evidence for the formation of dipoles at direct-contact HfO_2_/Si interfaces. It is proposed that Si-O bonds of about one monolayer at the HfO_2_/Si interface naturally lead to large differences in the electrostatic potential due to the large dielectric constant of HfO_2_. It was also found that dipole scattering in direct-contact HfO_2_/Si MOSFETs is not of major concern in terms of the mobility criterion.
